# The Norwegian Adaptation of the Big Five Inventory-2

**DOI:** 10.3389/fpsyg.2022.858920

**Published:** 2022-05-18

**Authors:** Hallvard Føllesdal, Christopher J. Soto

**Affiliations:** ^1^Department of Organizational Behaviour, BI Norwegian Business School, Oslo, Norway; ^2^Department of Psychology, Colby College, Waterville, ME, United States

**Keywords:** BFI-2, personality, big five, empathy, psychometric, personality assessment, empathic concern, perspective taking

## Abstract

Two studies were conducted to assess the psychometric properties of scores from the Norwegian adaptation of the Big Five Inventory-2 (BFI-2). In Study 1, the BFI-2 was translated to Norwegian and the scores from a convenience sample (*N* = 606) demonstrated good psychometric properties. BFI-2 scores from subsamples correlated in expected ways with self- and other ratings of the Big Five, and with self-ratings of empathic concern and perspective taking. In Study 2, after some minor improvements in translation, the psychometric properties of BFI-2 scores were assessed in a new sample (*N* = 409). Results from random intercept EFA of scores supported the proposed model. The psychometric properties of two shorter versions of the inventory, the BFI-2-S and BFI-2-XS, were also examined. Overall, the results suggest that the Norwegian adaptation of the BFI-2 provide reliable and valid scores.

## Introduction

The Big Five model of personality describes five broad personality domains, often termed Extraversion, Agreeableness, Conscientiousness, Neuroticism, and Openness (John and Srivastava, 1999). Each domain encompasses a broad group of personality traits, or relatively stable patterns of thinking, feeling, and behaving. In personality research, the Big Five Inventory (BFI; John and Srivastava, 1999) has become one of the most frequently used measures of Big Five. Recently, a new version of this inventory was developed: the Big Five Inventory-2 (BFI-2; Soto and John, 2017a). The BFI-2 is freely available for use in research, and has been adapted to several languages, which may facilitate personality research around the world. In the present research, we will assess whether the good psychometric properties of scores from the original BFI-2 extend to the Norwegian adaptation of the BFI-2.

Compared to the original BFI, the BFI-2 has been improved in several ways (for a thorough description, see Soto and John, 2017a). First, for each Big Five domain, the BFI-2 measures three facets that frequently appear in various personality hierarchies. One facet is considered factor pure, in that “previous research has identified [it] as central to its own domain and independent from the other four domains” (Soto and John, 2017a, p. 5). The two other facets are complementary facets, which means that they “are prominent in the personality literature and represented in the original BFI’s item content” (Soto and John, 2017a, p. 5). Previous research has shown that the inclusion of facet-level traits can improve the descriptive and predictive power of personality inventories (Hofstee, 1992; Hendriks et al., 1999; Paunonen and Ashton, 2001; Ashton et al., 2014). Second, to control for acquiescence, the items in each scale are content-balanced, that is, they consist of an equal number of positively and negatively keyed items. Third, new labels were introduced for two of the domains to better reflect their content: Negative Emotionality (instead of Neuroticism) and Open-Mindedness (instead of Openness). Each of the Big Five personality domains is measured by three facets, which are measured by four items each (i.e., 12 items per domain). Overall, the BFI-2 consists of 60 items, where 18 items are identical to items in the original BFI, 14 are revised BFI items, and 28 are entirely new items.

Many studies utilizing various adaptations of the BFI-2 into different languages have provided strong support for the validity of BFI-2 scores. For instance, several studies have supported the convergent and divergent validity of scores (provided by either the full or brief versions), by relating them to a range of other personality inventories, like the BFAS, NEO PI-R, NEO-FFI, Big Five Mini-Markers, and peer ratings of the BFI-2 in English (Soto and John, 2017a); the NEO PI-R in German (Rammstedt et al., 2018); the Big Five Mini-Markers in Danish (Vedel et al., 2021); the IPIP Big Five scales in Dutch (Denissen et al., 2019); and the Big Five factor markers in Russian (Shchebetenko et al., 2019). Studies have also demonstrated that facet scores may outperform broad domain scores in the prediction of various criteria, like behavioral and psychological outcomes (Soto and John, 2017a); affective states, self-endorsed values, and satisfaction with life outcomes (Denissen et al., 2019); and educational attainment, income, health, and life satisfaction (Danner et al., 2021). Thus, a large body of evidence supports the validity of scores provided by various adaptations of the BFI-2 into different languages.

The aim of the present research was to develop a Norwegian adaptation of the BFI-2 and assess the psychometric properties of scores. In Study 1, the BFI-2 was translated to Norwegian and the psychometric properties of scores assessed. In Study 2, the good psychometric properties of BFI-2 scores were confirmed in a new sample, after some minor improvements in translation.

## Study 1

The aim of Study 1 was to translate the BFI-2 into Norwegian and assess the psychometric properties of scores. That is, we wanted to assess the factor structure and reliability of scores; their convergent and divergent validity in relation to self- and others’ ratings of the Big Five; and their predictive validity in relation to empathic concern and perspective taking, two constructs that have been frequently used as indicators of empathy (Davis, 1980, 1983).

While few studies have looked at how empathic concern and perspective taking are related to facets of Big Five, several studies have examined their relationship with the broad Big Five domains (Graziano et al., 2007; Mooradian et al., 2011; Habashi et al., 2016; Melchers et al., 2016; Neumann et al., 2016; Song and Shi, 2017; Guilera et al., 2019). These studies found that empathic concern was positively and strongly related to agreeableness (*r* = 0.31–0.63); but also to extraversion (*r* = 0.05–0.29), openness (*r* = 0.04–0.22), conscientiousness (*r* = 0.05–0.22); and neuroticism (*r* = −0.04 to 0.17). Perspective taking on the other hand, was positively related to agreeableness (*r* = 0.22–0.43), openness (*r* = 0.20–0.30), conscientiousness (*r* = 0.11–0.28), and extraversion (*r* = 0.00–0.15), while negatively related to neuroticism (*r* = −0.13 to −0.33).

Based on these findings, we expect that empathic concern should be positively related to several Big Five domains, especially agreeableness and extraversion. Perspective taking should also be positively related to several Big Five domains, but negatively related to negative emotionality. In the present study, we will also examine whether the BFI-2 facets may outperform the broad domains in predicting these constructs.

### Materials and Methods

#### Participants and Procedure

A convenience sample (*N* = 601) was used, consisting of 425 participants from an executive education program at a Norwegian business school, ranging in age from 25 to 65 years old (*M* = 42.22, *SD* = 7.39), mainly consisting of women (72.2% women, 25.4% men, 2.4% did not report gender, no difference in mean age between men and women); 28 Master of Science students in an organizational psychology class at a Norwegian business school, ranging from 22 to 39 years (*M* = 25.36, *SD* = 0.44, gender not reported to preserve anonymity); and 148 sales employees from a Norwegian company (neither age nor sex were reported in order to preserve anonymity). The students completed the BFI-2 in a paper and pencil version, while sales employees completed an online version. Participants and colleagues also completed other questionnaires not reported on here.

#### Measures

##### Big Five Inventory-2

The process of translating the BFI-2 into Norwegian was conducted in collaboration with the authors of the original BFI-2, who had developed detailed translation guidelines for this purpose. As some BFI-2 items are identical with items in the BFI, the translation was informed by results from a principal component analysis (PCA) of item scores from the Norwegian adaptation of the BFI (Engvik and Føllesdal, 2005) in a sample of 1,767 Norwegians (H. Engvik, personal communication, July 1, 2016). The factor loadings indicated that some item translations might be improved. The BFI-2 items were translated to Norwegian by the principal author in cooperation with a translator, and back-translated to English by a bilingual psychologist, and the final translation was reviewed by the authors of the original BFI-2. Informed by the BFI-2 authors’ experiences with adapting this measure into other languages, and the results from the PCA of item scores from the Norwegian adaptation of BFI, alternative translations of six items were included and tested out in the first version of the inventory. For instance, results from PCA of BFI data indicated that the translation of one of the items (“Er selvhevdende” [“Has an assertive personality”]) loaded most strongly (and negatively) on Agreeableness, rather on Extraversion which was the intended domain. An alternative translation of this item was therefore included (“Er selvsikker, gjør seg gjeldende”). Likewise, alternative translations of five other items were included to be tested out. A five-point Likert scale was used, with the labels *helt uenig* [totally disagree], *litt uenig* [somewhat disagree], *nøytral/ingen oppfatning* [neutral/no opinion], *litt enig* [somewhat agree], and *helt enig* [totally agree].

##### Big Five Inventory

Five days after completing the BFI-2, a subsample of executive students (*n* = 209) completed the 44-item Norwegian adaptation of BFI (John and Srivastava, 1999), as part of a larger research project. We utilized these data to obtain preliminary evidence for the convergent and divergent validity of BFI-2 scores. Although the BFI is the precursor of the BFI-2, only about one-third of the items are identical to items in the BFI, while two-thirds of the items in BFI-2 are new or revised BFI items. In Norway, the BFI is frequently used to measure the Big Five, and it has been found to provide scores with good psychometric properties (Engvik and Føllesdal, 2005). A 7-point rating scale was used (which is standard in the Norwegian adaptation of BFI), ranging from *helt uenig* [totally disagree] to *helt enig* [totally agree], with no labels for the scale points in between. In the present sample, Cronbach’s alpha for the domain scores were 0.84 (Extraversion), 0.73 (Agreeableness), 0.78 (Conscientiousness), 0.82 (Neuroticism), and 0.84 (Openness).

##### Big Five Inventory-20

A subsample of executive students (*n* = 279) were also rated on the BFI-20 by an average of 4.5 colleagues (*n* = 1246) at work (the participants worked in different organizations, and each participant recruited raters among their colleagues (supervisor, subordinates, and same level colleagues). These data were also collected as part of a larger research project but utilized in the present study to provide preliminary evidence of convergent and divergent validity of BFI-2 scores. The BFI-20 is a brief 20-item version of the Norwegian adaptation of the BFI, which has been demonstrated to provide scores with adequate structural and predictive validity; and reliability coefficients in the range of 0.57–0.78 (Engvik and Clausen, 2011). Only four out of 20 items in the BFI-20 are identical to BFI-2 items, while six of the items in BFI-20 were slightly revised in the BFI-2. A 7-point rating scale was used, ranging from *helt uenig* [totally disagree] to *helt enig* [totally agree], with no labels for the scale points in between.

##### Empathic Concern and Perspective Taking

Immediately after completing the BFI-2, 220 of the participants completed two seven-item scales selected from the Interpersonal Reactivity Index (Davis, 1980). These data were also collected as part of a larger research project but utilized in the present study to examine the predictive validity of BFI-2 scores. The selected scales, Empathic Concern and Perspective Taking, are frequently used as measures of empathy. Empathic Concern “assesses ‘other-oriented’ feelings of sympathy and concern for unfortunate others” (Davis, 1983, p. 114), and an example item is “I often have tender, concerned feelings for people less fortunate than me.” Perspective Taking measures “the tendency to spontaneously adopt the psychological point of view of others” (Davis, 1983, p. 113–114), and an example item is “I believe that there are two sides to every question and try to look at them both.” A 5-point rating scale was used, ranging from *passer ikke* [inaccurate] to *passer helt* [*accurate*], with no labels for the scale points in between. Cronbach’s alpha in the present sample were 0.77 and 0.76 for Empathic Concern and Perspective Taking, respectively, and the two scale scores were only modestly correlated (*r* = 0.23, *p* = 0.000).

### Results and Discussion

Results from reliability analyses and item-total correlations of the 66 candidate items were used to select the final set of 60 items for the Norwegian BFI-2. After selecting this final item set, we conducted identical analyses to Soto and John (2017a) to be able to compare the psychometric properties of scores with those reported for the original, English-language BFI-2 (for an explanation of the rationale for the various analyses, see Soto and John, 2017a).

In order to assess the structure in the BFI-2 scores, a Principal Component Analysis (PCA) was performed. Although PCA may not be optimally suited to assess the latent structure in scores (Conway and Huffcutt, 2003), this type of analysis was chosen in order to enable comparison with results reported for the original BFI-2 (Soto and John, 2017a). PCA were conducted on item scores after within-person-centering, in order to control for acquiescence. That is, each item score for each person was centered around their within-person mean across all 60 items (for an explanation of this approach, see Soto and John, 2017a). PCA with varimax rotation, requesting five components, revealed that 59 of the 60 item scores had their highest loading on the intended Big Five component (Table 1). Moreover, a PCA with varimax rotation of the 15 facet scores (Table 2) showed that all facets had their highest loading on the intended Big Five domains. Cronbach’s alpha ranged from 0.79 to 0.86 for the domain scores (Table 3) and from 0.57 to 0.77 for the facet scores (Table 4). In order to assess the similarity of the principal components obtained in the present study with those reported for the original BFI-2 (i.e., for the Internet sample, Soto and John, 2017a, p. 12), Tucker’s factor congruence was estimated for pairs of corresponding components, using the R psych package (Revelle, 2021). A congruence coefficient above 0.95 indicates that components can be considered equal (Lorenzo-Seva and ten Berge, 2006). The coefficients were 0.96–0.97 for components derived from item scores, and 0.98–0.99 for components derived from the facets. These findings indicate that the components derived from scores from the Norwegian adaptation of the BFI-2 can be considered equivalent to corresponding components in the original BFI-2. Overall, the psychometric properties are highly similar to those reported for the original BFI-2, and the structural validity of scores was supported.

**TABLE 1:** Loadings from a principal component analysis of the 60 within-person centered Norwegian BFI-2 items (Study 1 and 2).

BFI-2 item			E	A	C	N	O	Complexity
Extraversion								
	Sociability							
		cbfi1	**0.70/0.61**	0.28/0.32	0.01/-0.01	0.06/0.08	0.00/0.03	1.32/1.56
		cbfi16	**–0.73/–0.59**	0.04/0.01	0.07/0.14	0.04/0.03	0.00/0.06	1.03/1.14
		cbfi31	**–0.66/–0.70**	0.00/–0.05	0.03/–0.04	–0.23/–0.14	–0.01/–0.07	1.24/1.12
		cbfi46	**0.72/0.65**	0.14/0.25	–0.01/0.03	–0.06/–0.08	0.04/0.05	1.09/1.34
	Assertiveness							
		cbfi6	**0.54/0.58**	–0.13/–0.15	0.13/0.12	0.36/0.42	0.16/0.14	2.25/2.23
		cbfi21	**0.53/0.68**	–0.35/–0.17	0.06/0.08	0.06/0.12	0.13/0.13	1.92/1.30
		cbfi36	**–0.45/–0.51**	0.11/0.08	–0.07/0.04	–0.20/–0.13	–0.18/–0.18	1.95/1.46
		cbfi51	**–0.31/–0.57**	0.26/0.19	–0.19/–0.09	–0.10/–0.08	–0.24/–0.30	3.90/1.88
	Energy level							
		cbfi11	**–0.48/–0.57**	–0.20/–0.32	–0.06/–0.06	0.12/0.10	–0.25/–0.13	2.12/1.80
		cbfi26	–0.21**/–0.30**	–0.04/0.05	**–0.24**/–0.26	–0.07/–0.18	–0.22/–0.06	3.23/2.80
		cbfi41	**0.54/0.59**	0.18/0.23	0.27/0.18	0.23/0.29	0.17/0.01	2.42/2.03
		cbfi56	**0.63/0.58**	0.22/0.32	0.19/0.06	0.04/0.00	0.24/0.16	1.76/1.76
Agreeableness								
	Compassion							
		cbfi2	0.07/0.09	**0.68/0.71**	0.05/–0.02	–0.14/–0.24	0.13/0.05	1.20/1.27
		cbfi17	–0.04/–0.17	**–0.50/–0.60**	–0.06/0.08	0.08/0.14	–0.15/–0.09	1.28/1.37
		cbfi32	–0.01/0.09	**0.54/0.57**	0.24/0.20	0.04/0.03	0.07/0.12	1.44/1.40
		cbfi47	–0.10/–0.13	**–0.66/–0.70**	–0.08/–0.08	0.07/0.15	–0.05/0.00	1.11/1.19
	Respectfulness							
		cbfi7	0.03/–0.13	**0.50/0.52**	0.32/0.30	0.16/0.10	0.06/0.04	2.00/1.85
		cbfi22	0.33/0.33	**–0.43/–0.40**	–0.06/–0.15	–0.10/–0.08	0.11/0.09	2.22/2.46
		cbfi37	0.12/0.16	**–0.48/–0.55**	–0.27/–0.31	–0.19/–0.21	–0.01/–0.03	2.09/2.13
		cbfi52	–0.11/–0.11	**0.56/0.57**	0.29/0.20	0.11/0.09	0.05/–0.02	1.71/1.38
	Trust							
		cbfi12	0.00/0.02	**–0.49/–0.51**	–0.07/–0.04	–0.22/–0.30	–0.04/–0.10	1.46/1.73
		cbfi27	0.04/0.09	**0.52/0.51**	–0.02/0.00	0.16/0.22	0.12/0.07	1.32/1.47
		cbfi42	–0.23/–0.17	**–0.50/–0.45**	0.01/–0.01	–0.23/–0.31	0.06/0.12	1.91/2.28
		cbfi57	0.15/0.16	**0.58/0.62**	0.09/–0.01	0.20/0.20	–0.01/–0.05	1.44/1.37
Conscientiousness								
	Organization							
		cbfi3	0.06/0.01	0.19/0.08	**–0.72/–0.74**	0.00/0.00	0.10/0.08	1.19/1.05
		cbfi18	–0.04/–0.12	–0.03/0.01	**0.78/0.73**	0.00/–0.15	–0.03/–0.03	1.01/1.14
		cbfi33	0.01/–0.08	0.15/0.07	**0.69/0.74**	0.00/–0.06	–0.03/0.00	1.10/1.05
		cbfi48	–0.07/0.00	–0.08/–0.07	**–0.54/–0.57**	0.05/0.08	0.04/–0.03	1.12/1.08
	Productiveness							
		cbfi8	–0.16/–0.19	–0.10/–0.06	**–0.60/–0.61**	0.00/–0.15	–0.06/–0.01	1.22/1.35
		cbfi23	–0.10/–0.27	–0.05/–0.02	**–0.56/–0.51**	–0.10/–0.17	–0.08/–0.01	1.19/1.78
		cbfi38	0.20/0.33	0.03/0.07	**0.59/0.57**	0.10/0.17	0.08/0.01	1.35/1.85
		cbfi53	0.01/0.12	0.23/0.11	**0.59/0.55**	0.09/0.23	0.12/0.05	1.44/1.56
	Responsibility							
		cbfi13	0.04/–0.08	0.27/0.29	**0.45/0.55**	0.23/0.05	0.05/–0.10	2.27/1.67
		cbfi28	0.03/0.00	–0.11/–0.02	**–0.46/–0.67**	–0.06/0.00	0.09/–0.11	1.24/1.06
		cbfi43	0.10/0.04	0.35/0.36	**0.48/0.46**	0.14/0.13	0.04/0.05	2.17/2.12
		cbfi58	0.09/0.08	–0.17/–0.18	**–0.51/–0.67**	–0.07/–0.09	0.05/0.02	1.36/1.21
Negative Emotionality								
	Anxiety							
		cbfi4	–0.02/0.06	0.01/–0.06	0.02/0.02	**0.67/0.69**	0.13/0.02	1.08/1.03
		cbfi19	–0.19/–0.16	–0.05/–0.04	0.14/0.07	**–0.64/–0.66**	–0.05/–0.03	1.29/1.15
		cbfi34	–0.16/–0.14	0.00/0.06	0.05/0.09	**–0.74/–0.76**	–0.02/0.02	1.11/1.11
		cbfi49	0.09/0.14	–0.02/–0.06	–0.01/–0.01	**0.59/0.72**	0.03/–0.01	1.06/1.09
	Depression							
		cbfi9	0.09/0.16	0.20/0.22	0.10/0.13	**0.38/0.56**	0.23/0.10	2.55/1.69
		cbfi24	0.27/0.32	0.13/0.04	0.23/0.13	**0.53/0.59**	0.03/0.10	2.07/1.74
		cbfi39	–0.24/–0.18	–0.09/–0.13	–0.09/–0.06	**–0.64/–0.79**	0.02/0.11	1.36/1.21
		cbfi54	–0.27/–0.22	–0.09/–0.22	–0.16/–0.09	**–0.65/–0.75**	0.00/0.07	1.55/1.41
	Emotional Volatility							
		cbfi14	–0.04/0.07	–0.23/–0.15	–0.12/–0.11	**–0.59/–0.65**	–0.06/–0.07	1.43/1.22
		cbfi29	–0.15/–0.14	0.06/0.04	0.12/0.04	**0.75/0.83**	–0.09/–0.02	1.17/1.07
		cbfi44	–0.21/–0.09	0.09/0.04	0.19/0.14	**0.60/0.71**	0.00/0.01	1.52/1.12
		cbfi59	0.26/0.32	–0.16/–0.18	–0.06/–0.12	**–0.51/–0.54**	0.10/–0.01	1.84/2.01
Open-Mindedness								
	Aesthetic Sensitivity							
		cbfi5	0.01/–0.02	–0.01/–0.06	0.02/0.07	0.10/0.10	**–0.67/–0.60**	1.05/1.11
		cbfi20	0.04/–0.08	0.02/0.05	–0.11/0.05	–0.07/–0.02	**0.59/0.70**	1.11/1.05
		cbfi35	0.03/0.02	0.14/0.09	0.01/0.04	–0.09/–0.07	**0.63/0.70**	1.15/1.06
		cbfi50	–0.08/–0.09	–0.21/–0.14	0.02/0.00	0.10/0.14	**–0.51/–0.63**	1.49/1.25
	Intellectual Curiosity							
		cbfi10	0.16/0.21	0.14/0.23	0.05/0.06	0.12/0.11	**0.48/0.51**	1.60/1.92
		cbfi25	–0.18/–0.06	0.07/0.16	0.06/0.04	–0.08/–0.12	**–0.46/–0.47**	1.45/1.43
		cbfi40	–0.06/–0.17	0.06/–0.01	0.09/0.02	0.01/–0.20	**0.52/0.51**	1.12/1.55
		cbfi55	–0.05/–0.05	–0.03/0.03	0.08/0.02	–0.09/–0.12	**–0.61/–0.58**	1.09/1.11
	Creative Imagination							
		cbfi15	0.07/0.19	–0.06/–0.09	0.07/0.24	0.16/0.25	**0.55/0.56**	1.27/2.14
		cbfi30	–0.07/–0.22	–0.04/0.01	–0.02/0.05	–0.01/–0.05	**–0.69/–0.66**	1.03/1.25
		cbfi45	–0.06/–0.15	–0.04/–0.09	–0.10/0.02	–0.02/0.02	**–0.54/–0.44**	1.11/1.33
		cbfi60	0.14/0.32	–0.03/–0.03	–0.04/0.03	0.13/0.23	**0.60/0.62**	1.23/1.82
Mean item complexity								1.55/1.50

*E, Extraversion; A, Agreeableness; C, Conscientiousness; N, Negative Emotionality; O, Open-Mindedness; Complexity, Hofman’s complexity index (Hofmann, 1978). Values before and after the forward slash are from Study 1 and 2, respectively. The strongest loading for each item is bolded.*

**TABLE 2:** Loadings from a principal component analysis of scores from the 15 BFI-2 facets (Study 1 and 2).

BFI-2 facets	E	A	C	N	O
Extraversion					
Sociability	**0.88/0.83**	0.10/0.15	–0.07/–0.05	–0.04/–0.01	0.01/0.03
Assertiveness	**0.67/0.75**	–0.30/–0.20	0.12/0.04	–0.24/–0.21	0.22/0.24
Energy level	**0.68/0.77**	0.22/0.23	0.25/0.18	–0.04/–0.10	0.29/0.12
Agreeableness					
Compassion	0.07/0.20	**0.80/0.83**	0.14/0.06	0.09/0.19	0.15/0.07
Respectfulness	–0.19/–0.23	**0.69/0.72**	0.30/0.37	–0.22/–0.20	–0.01/–0.02
Trust	0.18/0.19	**0.75/0.75**	0.03/0.01	–0.28/–0.37	0.03/0.01
Conscientiousness					
Organization	0.00/–0.04	0.00/0.02	**0.86/0.84**	0.01/0.08	–0.08/–0.02
Productiveness	0.22/0.34	0.14/0.03	**0.81/0.73**	–0.09/–0.22	0.10/0.03
Responsibility	–0.02/–0.01	0.29/0.22	**0.70/0.83**	–0.17/–0.08	–0.02/0.03
Negative Emotionality					
Anxiety	–0.16/–0.18	–0.01/0.05	0.06/0.05	**0.85/0.86**	–0.07/–0.01
Depression	–0.31/–0.31	–0.17/–0.16	–0.18/–0.12	**0.74/0.82**	–0.08/–0.01
Emotional Volatility	0.16/0.15	–0.16/–0.12	–0.16/–0.13	**0.83/0.86**	0.04/–0.03
Open-mindedness					
Aesthetic Sensitivity	0.11/0.05	0.03/–0.02	–0.02/–0.02	–0.12/–0.05	**0.79/0.82**
Intellectual Curiosity	0.05/0.02	0.12/0.12	–0.05/0.00	0.12/0.13	**0.75/0.81**
Creative Imagination	0.15/0.30	0.00/–0.04	0.05/0.06	–0.08/–0.14	**0.78/0.75**

*E, Extraversion; A, Agreeableness; C, Conscientiousness; N, Negative Emotionality; O, Open-Mindedness. Values before and after the forward slash are from Study 1 and 2, respectively. The strongest loading for each item is bolded.*

**TABLE 3:** Reliability estimates (with confidence intervals) and intercorrelations for scores from BFI-2 domains (Study 1 and 2).

Domain/Facet	α [95% CI]	*M*	*SD*	E	A	C	N	O
Extraversion	0.82 [0.80, 0.84]/0.85 [0.83, 0.87]	3.80/3.57	0.58/0.66		0.11/0.18	0.18/0.18	–0.25/–0.26	0.31/0.30
Agreeableness	0.79 [0.76, 0.81]/0.81 [0.78, 0.83]	4.00/3.76	0.51/0.58	0.11/0.18		0.34/0.29	–0.30/–0.25	0.14/0.10
Conscientiousness	0.83 [0.80, 0.85]/0.85 [0.83, 0.87]	4.22/3.84	0.53/0.64	0.18/0.18	0.34/0.28		–0.21/–0.18	0.01/0.10
Negative Emotionality	0.86 [0.84, 0.87]/0.90 [0.89, 0.92]	2.25/2.65	0.67/0.86	–0.25/–0.26	–0.30/–0.25	–0.21/–0.18		–0.08/–0.10
Open-mindedness	0.81 [0.79, 0.84]/0.83 [0.80, 0.85]	3.74/3.51	0.64/0.71	0.31/0.30	0.14/0.10	0.01/0.10	–0.08/–0.10	

*E, Extraversion; A, Agreeableness; C, Conscientiousness; N, Negative Emotionality; O, Open-Mindedness. The figures before and after the forward slash are from Study 1 and 2, respectively. N = 599–601 for Study 1, and N = 409 for Study 2. Absolute correlations 0.11 or stronger (for Study 1) and 0.18 or stronger (for Study 2) are significant at p < 0.01. α [95% CI] = Cronbach’s alpha with 95% confidence intervals estimated with the two-way mixed consistency approach for estimating ICC in SPSS (Bravo and Potvin, 1991; Baumgartner and Chung, 2001).*

**TABLE 4:** Reliability, descriptive statistics, and intercorrelations for scores from BFI-2 facets (Study 1 and 2).

				E			A			C			N			O		
Facet	α [95% CI]	*M*	*SD*	Soc	Ass	Ene	Com	Rsp	Tru	Org	Pro	Res	Anx	Dep	Emo	Int	Aes	Cre
Sociability	0.77 [0.74, 0.80]/0.77 [0.73, 0.80]	3.66/3.43	0.85/0.86	-	**0.53**	**0.54**	0.22	–0.05	0.23	–0.02	0.17	0.03	–0.15	–0.25	0.01	0.06	0.12	0.25
Assertiveness	0.67 [0.63, 0.71]/0.77 [0.73, 0.80]	3.66/3.51	0.69/0.83	**0.48**	**–**	**0.46**	0.01	–0.20	0.09	–0.02	0.26	0.05	–0.31	–0.34	–0.07	0.22	0.15	0.42
Energy level	0.67 [0.62, 0.71]/0.64 [0.58, 0.70]	4.07/3.77	0.67/0.73	**0.48**	**0.32**	**–**	0.29	0.08	0.32	0.10	0.43	0.15	–0.20	–0.40	–0.02	0.18	0.13	0.30
Compassion	0.69 [0.64, 0.72]/0.75 [0.70, 0.79]	4.28/4.08	0.63/0.76	0.10	–0.06	0.20	**–**	**0.45**	**0.48**	0.06	0.11	0.23	0.12	–0.07	0.06	0.04	0.13	0.09
Respectfulness	0.57 [0.51, 0.63]/0.57 [0.49, 0.63]	4.03/3.85	0.58/0.62	–0.06	–0.16	0.12	**0.43**	**–**	**0.46**	0.26	0.24	0.46	–0.08	–0.21	–0.32	–0.06	0.01	–0.01
Trust	0.65 [0.60, 0.69]/0.67 [0.61, 0.72]	3.70/3.35	0.71/0.79	0.16	–0.01	0.27	**0.44**	**0.43**	**–**	0.06	0.20	0.16	–0.29	–0.44	–0.33	0.04	0.07	0.05
Organization	0.77 [0.74, 0.80]/0.82 [0.79, 0.85]	4.09/3.79	0.78/0.89	–0.02	0.05	0.18	0.11	0.24	0.08	**–**	**0.44**	**0.56**	0.09	–0.03	–0.07	–0.01	0.02	–0.04
Productiveness	0.65 [0.60, 0.70]/0.74 [0.70, 0.78]	4.27/3.75	0.61/0.78	0.12	0.19	0.41	0.22	0.30	0.22	**0.56**	**–**	**0.51**	–0.21	–0.37	–0.19	0.06	0.01	0.20
Responsibility	0.57 [0.51, 0.62]/0.67 [0.62, 0.72]	4.31/3.98	0.55/0.68	0.02	0.08	0.15	0.33	0.35	0.26	**0.47**	**0.48**	**–**	–0.02	–0.19	–0.21	–0.02	0.04	0.08
Anxiety	0.76 [0.72, 0.79]/0.82 [0.80, 0.85]	2.53/2.89	0.89/1.01	–0.17	–0.25	–0.17	0.01	–0.10	–0.27	0.03	–0.09	–0.10	**–**	**0.67**	**0.59**	–0.02	0.05	–0.19
Depression	0.71 [0.67, 0.75]/0.81 [0.78, 0.84]	1.89/2.40	0.69/0.96	–0.24	–0.31	–0.36	–0.13	–0.22	–0.37	–0.15	–0.29	–0.26	**0.58**	**–**	**0.64**	–0.10	0.06	–0.18
Emotional Volatility	0.73 [0.69, 0.76]/0.80 [0.77, 0.83]	2.34/2.66	0.84/1.02	0.02	–0.07	–0.02	–0.09	–0.37	–0.26	–0.12	–0.17	–0.28	**0.54**	**0.53**	**–**	–0.04	0.07	–0.10
Intellectual Curiosity	0.62 [0.57, 0.67]/0.57 [0.50, 0.64]	3.91/3.81	0.72/0.75	0.14	0.28	0.27	0.13	0.03	0.12	–0.06	0.08	0.02	–0.13	–0.14	–0.05	**–**	**0.46**	**0.48**
Aesthetic Sensitivity	0.77 [0.74, 0.80]/0.79 [0.75, 0.82]	3.37/3.08	1.02/1.08	0.11	0.08	0.24	0.16	–0.01	0.07	–0.05	0.04	–0.02	–0.01	–0.06	0.11	**0.42**	**–**	**0.46**
Creative Imagination	0.74 [0.70, 0.77]/0.75 [0.70, 0.78]	3.93/3.65	0.71/0.82	0.14	0.27	0.32	0.14	0.01	0.09	–0.04	0.15	0.02	–0.13	–0.13	–0.02	**0.47**	**0.38**	**–**

*The figures before and after the forward slash are from Study 1 and 2, respectively. Correlation coefficients below the diagonal are for Study 1, and correlations coefficients above the diagonal are for Study 2. Absolute correlations 0.11 or stronger (Study 1) and 0.15 or stronger (Study 2) are significant at p < 0.01. E, Extraversion; A, Agreeableness; C, Conscientiousness; N, Negative Emotionality; O, Open-Mindedness; Soc, Sociability; Ass, Assertiveness; Ene, Energy Level; Com, Compassion; Rsp, Respectfulness; Tru, Trust; Org, Organization; Pro, Productiveness; Res, Responsibility; Anx, Anxiety; Dep, Depression; Emo, Emotional Volatility; Int, Intellectual Curiosity; Aes, Aesthetic Sensitivity; Cre, Creative Imagination. Within-domain correlations are bolded. α [95% CI], Cronbach’s alpha with 95% confidence intervals estimated with the two-way mixed consistency approach for estimating ICC in SPSS (Bravo and Potvin, 1991; Baumgartner and Chung, 2001).*

#### Convergent and Divergent Validity

As mentioned previously, both self–ratings with the BFI and other ratings with the BFI-20 were collected for a subsample of executive students as part of a larger research project. In the present study, these data were utilized to provide preliminary evidence of convergent and divergent validity of scores from the Norwegian adaptation of BFI-2. The results are presented in Table 6. First, we examined the correlations between BFI-2 scores and self-ratings of personality with the BFI. The BFI-2 domain scores were strongly related to corresponding self-rated BFI domain scores, with correlation coefficients ranging from 0.72 to 0.83, averaging 0.77. Moreover, the BFI-2 domain scores were weakly related to non-corresponding self-rated BFI domain scores, with correlations ranging from −0.25 to 0.24, and absolute correlations averaging 0.13. The average correlation between corresponding domains of 0.77 was somewhat lower than the corresponding average correlation of 0.92 reported by Soto and John (2017a). In their study, however, the BFI and BFI-2 were administered together, while in the present study these questionnaires were administered 5 days apart, which may have attenuated the correlations between the scores. Moreover, in the present study, a 7-point scale was used with the BFI, with rating labels on the endpoints only, which is standard for the Norwegian adaptation of the BFI.

Next, we examined the correlations between BFI-2 scores and colleagues’ ratings with the BFI-20. Due to the nested nature of data (raters nested within participants), a multilevel model was specified and analyzed using Mplus 8.7 using manifest personality scores. The intraclass correlations (ICC = 0.27–0.44) indicated that a substantial amount of variance in personality ratings was due to differences among rated targets, supporting the decision to use a multilevel model. The relationships between self-ratings and colleagues’ ratings of personality were assessed on the between-group level in the model, as colleagues are nested within participants. The results are presented in Table 5. As expected, the self-rated BFI-2 domain scores correlated most strongly with the corresponding domain scores rated by colleagues (average *r* = 0.47, range = 0.38–0.53), and weaker with the non-corresponding domains rated by colleagues (average |*r*| = 0.13, range −0.27 to 0.32). Overall, the pattern of correlations between self and other ratings of Big Five supports the convergent and divergent validity of BFI-2 scores.

**TABLE 5:** BFI-2 scores and their correlations with self-ratings on BFI and colleagues’ ratings on BFI-20.

	Self-report BFI*^a^*/Colleagues’ ratings, BFI-20*^b^*									
	Extraversion		Agreeableness		Conscientiousness		Neuroticism		Openness	
BFI-2 domains/facets	S	C	S	C	S	C	S	C	S	C
Extraversion	0.83**	0.53**	0.05	–0.02	0.05	−0.25**	−0.20**	−0.22**	0.22**	0.32**
Sociability	0.82**	0.60**	–0.02	0.03	–0.09	−0.27**	–0.10	−0.17**	0.14*	0.32**
Assertiveness	0.55**	0.25**	−0.15*	−0.17*	–0.02	–0.14	−0.19**	−0.22**	0.23**	0.24**
Energy level	0.53**	0.35**	0.30**	0.08	0.30**	−0.15*	−0.22**	–0.12	0.24**	0.20**
Agreeableness	0.04	0.07	0.72**	0.38**	0.21**	0.05	−0.25**	–0.10	–0.09	–0.08
Compassion	0.11	0.08	0.51**	0.38**	0.11	0.04	0.01	0.05	0.09	0.02
Respectfulness	−0.18*	–0.11	0.55**	0.30**	0.35**	0.17*	−0.19**	–0.11	−0.18**	−0.18**
Trust	0.13	0.16**	0.60**	0.22**	0.06	–0.08	−0.35**	–0.14	–0.11	–0.01
Conscientiousness	0.03	−0.20**	0.24**	0.18*	0.76**	0.52**	−0.23**	–0.15	−0.23**	−0.31**
Organization	0.01	−0.20**	0.13	0.18*	0.69**	0.53**	−0.16*	–0.10	−0.19**	−0.27**
Productiveness	0.07	–0.08	0.22**	0.10	0.65**	0.31**	−0.23**	−0.20**	−0.14*	−0.16*
Responsibility	–0.02	−0.20**	0.26**	0.17*	0.48**	0.40**	−0.18**	–0.01	−0.26**	−0.31**
Negative Emotionality	–0.09	–0.05	−0.19**	–0.05	–0.04	0.04	0.75**	0.51**	0.10	0.06
Anxiety	–0.11	–0.10	–0.04	0.10	0.17*	0.21**	0.60**	0.44**	0.02	–0.08
Depression	−0.24**	−0.17**	−0.23**	–0.09	−0.16*	0.03	0.66**	0.44**	0.09	0.02
Emotional Volatility	0.10	0.12	−0.20**	–0.11	–0.13	–0.11	0.55**	0.39**	0.14	0.19**
Open-Mindedness	0.13	0.14*	0.10	0.01	–0.13	−0.21**	0.00	–0.11	0.80**	0.39**
Intellectual Curiosity	0.16*	0.03	0.09	0.10	–0.11	–0.06	–0.10	−0.17*	0.57**	0.24**
Aesthetic Sensitivity	0.07	0.12	0.09	–0.03	–0.08	−0.17*	0.09	–0.08	0.64**	0.27**
Creative Imagination	0.08	0.17*	0.04	–0.03	–0.13	−0.27**	0.01	0.00	0.65**	0.40**
ICC Big Five Ratings		0.443		0.266		0.303		0.271		0.346

*S, Self-report on BFI; C, Colleagues ratings on BFI-20. For each BFI-2 domain and facet, the strongest correlation with each Big Five domain is bolded.*

*^a^N = 209. ^b^N = 279/1,246 participants/raters. ^c^N = 220.*

*p < 0.05. **p < 0.01.

#### Predicting Empathic Concern and Perspective Taking

To further assess the validity of scores, we examined how the BFI-2 facet and domain scores could predict self-ratings of Empathic Concern and Perspective Taking, and whether the BFI-2 facet scores could outperform the broad domains. As facets generally provide scores with lower reliability than domain scores (due to fewer items) it may be challenging to compare their predictive validity. Therefore, both facet and domain scores were corrected for measurement error by modeling them as latent variables in Mplus, by specifying the residual variance for each variable *x* to variance*_*x*_* × (1 − reliability*_*x*_*), based on the estimated reliability of scores in the present sample (Bollen, 1989). The results are presented in Table 6; in the following text, the results for the corrected (latent) variable scores are reported.

**TABLE 6:** Self-ratings of BFI-2 and correlations with empathic concern and perspective taking.

	Empathic Concern		Perspective Taking	
BFI-2 domains/facets	*r*	*r_c_*	*r*	*r_c_*
Extraversion	0.12	0.13	0.12	0.14
Sociability	0.14	0.16	0.05	0.06
Assertiveness	–0.03	–0.03	0.12	0.14
Energy level	0.15*	0.18*	0.14*	0.17*
Agreeableness	0.36**	0.40**	0.38**	0.43**
Compassion	0.59**	0.71**	0.28**	0.33**
Respectfulness	0.14*	0.18*	0.30**	0.40**
Trust	0.12	0.15	0.30**	0.37**
Conscientiousness	0.03	0.04	0.13	0.14
Organization	–0.02	–0.03	0.06	0.07
Productiveness	0.08	0.10	0.10	0.13
Responsibility	0.04	0.05	0.17*	0.22*
Negative Emotionality	0.35**	0.37**	−0.18**	−0.20**
Anxiety	0.31**	0.36**	–0.13	−0.15*
Depression	0.19**	0.22**	−0.19**	−0.22**
Emotional Volatility	0.31**	0.36**	–0.13	−0.15*
Open-Mindedness	0.27**	0.30**	0.32**	0.35**
Intellectual Curiosity	0.17*	0.21*	0.35**	0.45**
Aesthetic Sensitivity	0.29**	0.34**	0.18**	0.20**
Creative Imagination	0.15*	0.18*	0.24**	0.28**
*M*		3.92		3.89
*SD*		0.60		0.58

*rc = correlations corrected for measurement error by specifying the residual variance for each variable x to variance_x_ x (1-reliabilityx).*

N = 220. *p < 0.05. **p < 0.01.

The pattern of correlations between BFI-2 domain scores and Empathic Concern were in line with previous studies. That is, among the Big Five domains, Empathic Concern correlated most strongly with Agreeableness (Graziano et al., 2007; Mooradian et al., 2011; Melchers et al., 2016; Neumann et al., 2016; Song and Shi, 2017; Guilera et al., 2019). This relationship, however, seems to be mostly driven by the facet Compassion, as this correlation (*r* = 0.71) was substantially higher than for overall Agreeableness (*r* = 0.40). This is reasonable, as Compassion and Empathic Concern are conceptually very similar constructs, and because Compassion is considered a factor-pure facet of Agreeableness (Soto and John, 2017a). Empathic Concern was also positively correlated with the domain scores for Negative Emotionality and Open-mindedness, in line with findings reported by Song and Shi (2017). Moreover, all facets within these domains correlated positively with Empathic Concern. For Extraversion, the correlation with Empathic Concern was not significant, in contrast to findings reported in previous studies (Mooradian et al., 2011; Melchers et al., 2016; Neumann et al., 2016; Guilera et al., 2019). By examining the Extraversion facets, however, the scores from Energy Level were positively and significantly correlated with Empathic Concern, which underscores the importance of measuring facets in addition to domain scores.

The BFI-2 facets also seem to outperform the broad domains in predicting Empathic Concern. For four of the Big Five domains (all except Negative Emotionality), the correlations with Empathic Concern were stronger for facet scores than for domain scores. Moreover, the Big Five explained 50% of the variance (*R*^2^_*Adj*_. = 0.498, *p* = 0.000) in Empathic Concern, with Extraversion (β = 0.20, *p* = 0.008), Agreeableness (β = 0.57, *p* = 0.000), Negative Emotionality (β = 0.55, *p* = 0.000), and Open-Mindedness (β = 0.17, *p* = 0.017) as significant predictors. However, when using only one facet score from each domain as predictors (the facet score from each domain that was most strongly, and significantly, correlated with Empathic Concern), the four facets (Energy Level, Compassion, Anxiety, and Aesthetic Sensitivity) explained 56% of the variance (*R*^2^_*Adj*_. = 0.555, *p* = 0.000), with Compassion (β = 0.61, *p* = 0.000) and Anxiety (β = 0.20, *p* = 0.001) as significant predictors. It is important to note, however, that such an analysis, where we select as predictors the facets that are most strongly correlated with the outcome, may capitalize on chance and inflate the estimated explained variance (Ones and Viswesvaran, 1996). Future studies should therefore try to replicate these findings.

Turning to Perspective Taking, the correlation pattern with BFI-2 domain scores was also in line with previous studies. The scores from Perspective Taking were most strongly correlated with Agreeableness, as has been found in previous studies (Mooradian et al., 2011; Melchers et al., 2016; Neumann et al., 2016; Song and Shi, 2017). In contrast to Empathic Concern (which correlated most strongly with one facet within Agreeableness), Perspective Taking correlated positively with all three facets within Agreeableness. Moreover, Perspective Taking was negatively correlated with Negative Emotionality and positively correlated with Open-mindedness, as found in previous studies (Mooradian et al., 2011; Melchers et al., 2016; Song and Shi, 2017). Perspective Taking was also uncorrelated with the domain scores of Extraversion and Conscientiousness, while positively correlated with one facet score within each of these domains (Energy Level and Responsibility, respectively).

The scores from BFI-2 facets also seem to outperform broad domains in predicting Perspective Taking. That is, for all domains, except Agreeableness, the facet scores provided higher correlations than the domain scores. Regression analyses revealed that the Big Five explained 28% of the variance (*R*^2^_*Adj*_. = 0.283, *p* = 0.000) with Agreeableness (β = 0.21, *p* = 0.000) and Open-Mindedness (β = 0.20, *p* = 0.000) as the only significant predictors. When using only the one strongest facet from each domain as a predictor, the five facets (Energy Level, Respectfulness, Responsibility, Depression, and Intellectual Curiosity) together explained 41% of the variance (*R*^2^_*Adj*_. = 0.39, *p* = 0.000), with Respectfulness (β = 0.37, *p* = 0.001) and Intellectual Curiosity (β = 0.55, *p* = 0.000) as significant predictors.

Overall, the results suggest that the BFI-2 scores predict empathic concern and perspective taking in expected ways, supporting the construct validity of BFI-2 scores. Moreover, facet scores seem to be more important predictors than domain scores, but this pattern was not entirely consistent. For instance, the facets Compassion and Intellectual Curiosity outperformed their respective broad domains (Agreeableness and Open-Mindedness) in predicting Empathic Concern and Perspective Taking, respectively. For Negative Emotionality and Agreeableness, however, the domain scores outperformed the respective facet scores in predicting Empathic Concern and Perspective Taking, respectively. This illustrates that facets may be more important than domain scores in some instances, and not in others, which might be due to the degree of conceptual correspondence between predictor and criterion (for a discussion, see e.g., Judge et al., 2013). Overall, however, a faceted approach may be important in informing us about which aspects of personality are important for understanding and predicting empathy.

Taken together, the results of Study 1 suggest that the Norwegian adaptation of the BFI-2 provides scores with good psychometric properties. The proposed factor structure was supported, and the scales provided scores with adequate reliability, which correlated as expected with both self- and other ratings of the Big Five. Moreover, the scores correlated as expected with self-ratings of empathic concern and perspective taking. Finally, the facet scores generally outperformed the broad domain scores in predicting empathic concern and perspective taking. Overall, these findings support the construct validity of the BFI-2 scores.

Some minor issues, however, may be noted. First, while completing the BFI-2, some younger students reported that they did not understand the meaning of one of the words in item 28 [“skjødesløs” (careless)]. Thus, one may question the validity of scores from this scale in younger samples. Second, a closer look at the distribution of item scores revealed that item 13 provided scores with a relatively high mean (4.78) and a very large kurtosis (11.05), which is not optimal. Third, for 25 of the 60 items, the modal value was identical to the endpoints of the rating scale (either 1 or 5), suggesting that these items provide extreme scores and may not optimally differentiate among individuals. These issues were addressed in Study 2.

## Study 2

The aim of Study 2 was to assess the psychometric properties of scores from the Norwegian adaptation of BFI-2 in a new sample, after some slight improvements based on findings in Study 1. First, small revisions of items 13 and 28 were tested out in smaller samples before a final translation was selected for inclusion in the Norwegian adaptation of the BFI-2. That is, item 13 was rephrased from “Er til å stole på, stødig” to “Er pliktoppfyllende, gjør som avtalt” and item 28 was rephrased from “Kan være litt skjødesløs” to “Kan vaere litt slurvete, likeglad.” Second, in order to reduce the extreme scores on some of the items, the endpoint labels on the rating scale were rephrased to be more similar in meaning to the labels in the original BFI-2. That is, in Study 1 the endpoint labels *helt uenig* [totally disagree] and *helt enig* [totally agree] were used. These labels are commonly used in Norwegian questionnaires, and are also used in the Norwegian adaptation of the BFI (Engvik and Føllesdal, 2005). One may question, however, whether these labels express the same strong levels of disagreement and agreement as the labels in the original BFI-2, i.e., *strongly disagree* and *strongly agree*. The labels were therefore rephrased to *svært uenig* [strongly disagree] and *svært enig* [strongly agree], respectively.

One aim of the present study was therefore to see if a slight improvement in the translation of the endpoints of the rating scale might lead to less extreme scale scores. A second aim was to try to replicate the good psychometric properties of the final Norwegian adaptation of BFI-2 in a new sample. A third aim was to examine the preliminary psychometric properties of two shorter versions of the inventory, the BFI-2-S and BFI-2-XS (Soto and John, 2017b).

### Materials and Methods

#### Participants and Procedure

Students in an organizational psychology class at a Norwegian business school were invited to participate. They were provided with an anonymous link to a Qualtrics questionnaire and were encouraged to also recruit friends to participate. Participants were offered a brief feedback on their BFI-2 domain scores. Respondents were excluded if they (a) had completed the test previously, (b) did not select the response “I have done my best to answer all questions,” or (c) used less than 4 min or more than 40 min to answer all items. The final sample (*N* = 409) consisted of 250 women and 159 men, ranging from 17 to 71 years of age (*M* = 26.77 years, *SD* = 10.86, no difference in mean age across gender). About 57 percent of the sample were students at the Norwegian business school, and about 75 percent reported they were full-time students.

#### Measures

Participants completed the Norwegian adaptation of the BFI-2, using a 5-point scale with the labels 1 = *svært uenig* [strongly disagree], 2 = *litt uenig* [disagree a little], 3 = *nøytral* [neutral], 4 = *litt enig* [agree a little], and 5 = *svært enig* [strongly agree].

### Results and Discussion

We first compared the distribution of item scores with those from Study 1, and then assessed the Norwegian BFI-2’s psychometric properties by conducting several analyses that were identical to the ones reported in the study with the original BFI-2 (Soto and John, 2017a), that is, PCA and confirmatory factor analysis (CFA). In addition, we also utilized random intercept exploratory factor analysis (RI-EFA; Aichholzer, 2014), which has been used to assess the structure of scores from the BFI-2-S and BFI-2-XS (Soto and John, 2017b), and the Russian adaptation of BFI-2 (Shchebetenko et al., 2019), within a latent variable framework that also controls for individual differences in response style.

#### Score Distribution

The distribution of scores was better than in Study 1. First, the scores from the slightly revised item 13 had a lower mean, compared to Study 1 (4.56 vs. 4.78), and a lower kurtosis (2.44 vs. 11.05). Second, the modal value was identical to the most extreme value for only 17 items, compared to 25 items in Study 1. Though we expected these improvements, we cannot conclusively determine whether they were due to the slight change in the rating scale, or due to differences in the samples. Some items do still provide extreme scores, but this is also seen with the original BFI-2, as descriptive statistics for a large representative American sample (Soto, 2021) indicate that some scales may provide high mean scores.

#### Results From Principal Component Analysis and Congruence Analysis

Once again, in order to compare the psychometric properties of the scores from the Norwegian BFI-2 with those reported in the original study on the English-language instrument, we ran identical analyses to those reported on by Soto and John (2017a). A PCA with centered item scores (in order to control for acquiescence) revealed that the 60 items (Table 1) and 15 facet scores (Table 2) all had their highest loadings on the intended Big Five factor. Cronbach’s alpha ranged from 0.81 to 0.90 for the domain scores (Table 3), and 0.57 to 0.82 for the facet scores (Table 4), which is comparable to those reported for the original BFI-2. Moreover, we estimated congruence coefficients comparing the component loadings with those reported for the original BFI-2 (i.e., for the Internet sample, Soto and John, 2017a, p. 12). These coefficients were in the range 0.96–0.97 for components derived from the BFI-2 items, and 0.98–0.99 for components derived from the facets, indicating that the components may be considered equal. Finally, congruence coefficients comparing the components from Study 1 and Study 2 (in the present research) were in the range of 0.95–0.98 for the item-derived components, and 0.98–0.99 for the facet-derived components, indicating that components may be considered equal across the two studies.

#### Facet Level Structure

In order to assess the structure of scores at the facet level within each domain, three different CFA models were tested on the 12 item scores, using Mplus 8.7 (Muthén and Muthén, 1998-2017). The MLR estimator in Mplus was used, providing maximum likelihood parameter estimates with standard errors and a chi-square statistic that are robust to non-normality. The three models were identical to three of the models tested in the original study with the BFI-2 (Soto and John, 2017a, p. 16), which makes it possible to compare results. The results are provided in Table 7. In the *single domain* model, all item scores within one domain were specified to load on one factor. This model obtained poor fit in all domains. In the *three facets* model, each factor was measured by four items, corresponding to the three facets in the domain, and the three factors were allowed to correlate. This model obtained an acceptable fit in three out of five domains. In the *three facets plus acquiescence* model, all items were in addition constrained to load 1 on an acquiescence factor, which was specified to be uncorrelated with the three substantive facet factors (Billiet and McClendon, 2000). This model obtained an acceptable fit in all five domains, which was also the case for the original BFI-2 (Soto and John, 2017a). In the present study, the inclusion of an acquiescence factor improved the model fit significantly in three of the five Big Five domains. Overall, the results indicate that the Norwegian BFI-2 items capture three facet traits within each Big Five domain, and also allow researchers to model individual differences in acquiescent responding (Billiet and McClendon, 2000).

**TABLE 7:** Fit indices for CFA models of item scores from the BFI-2 domains.

Model	χ^2^	*df*	CFI	RMSEA [90% C.I.]	*p*(Δχ^2^)
Extraversion					
Single domain	337.13	54	0.783	0.113 [0.102–0.125]	
Three facets	155.15	51	0.920	0.071 [0.058–0.084]	
Three facets plus acquiescence	152.25	50	0.921	0.071 [0.058–0.084]	0.159
Agreeableness					
Single domain	294.31	54	0.756	0.104 [0.093–0.116]	
Three facets	191.89	51	0.857	0.082 [0.070–0.095]	
Three facets plus acquiescence	132.14	50	0.917	0.063 [0.050–0.077]	<0.000
Conscientiousness					
Single domain	383.84	54	0.751	0.122 [0.111–0.134]	
Three facets	173.72	51	0.907	0.077 [0.064–0.089]	
Three facets plus acquiescence	135.73	50	0.935	0.065 [0.052–0.078]	<0.000
Negative Emotionality					
Single domain	370.57	54	0.846	0.120 [0.108–0.131]	
Three facets	135.13	51	0.959	0.064 [0.051–0.077]	
Three facets plus acquiescence	112.37	50	0.970	0.055 [0.042–0.069]	<0.000
Open-Mindedness					
Single domain	325.47	54	0.759	0.111 [0.099–0.123]	
Three facets	110.67	51	0.947	0.053 [0.040–0.067]	
Three facets plus acquiescence	109.46	50	0.947	0.054 [0.040–0.068]	0.307

*N = 409. df, degree of freedom; CFI, comparative fit index; RMSEA, root mean square error of approximation. p(Δχ^2^) = p-value for difference in χ^2^ between the models with and without acquiescence factor, estimated with the formula for MLR Chi-square difference testing (Muthén and Muthén, 2022).*

#### Hierarchical Structure

In order to examine the structure of scores at both the domain and facet levels simultaneously, we used the exploratory structural equation modeling (ESEM) framework in Mplus to test an RI-EFA model (Aichholzer, 2014). This model allowed each of the 60 Norwegian BFI-2 item scores to load on both (a) five CF-varimax-rotated exploratory factors (to represent the Big Five domains) and (b) a confirmatory factor representing acquiescent response style. All loadings on the acquiescence factor were constrained to equal 1, and this factor was not allowed to correlate with the five domain factors. In addition, we allowed correlated residuals among the four items within each of the 15 BFI-2 facet scales (i.e., correlated uniqueness; Marsh et al., 2010). Thus, this model was specified to simultaneously represent (a) the Big Five domains (using exploratory factors), (b) the 15 BFI-2 facets (using correlated uniqueness), and (c) acquiescent response style. The model was tested in Mplus with the MLR estimator and provided a good fit (Table 8), χ^2^ (1,389, *N* = 409) = 1984.18, *p* = 0.000, RMSEA = 0.032 [95% C.I. = 0.029–0.036], and CFI = 0.935. Moreover, all 60 items had their strongest loading on their intended domain factor, though one item loaded equally strong on two domain factors (Supplementary Table 1). These results replicate findings from the Russian adaptation of the BFI-2 (Shchebetenko et al., 2019) by showing that ESEM can be used to simultaneously model the domain-level and facet-level structure of the BFI-2.

**TABLE 8:** Fit indices for RI-EFA of scores from different models of the 60 item scores from the BFI-2.

Model	χ^2^	*df*	CFI	RMSEA [90% C.I.]
Five factors	3220.90	1480	0.811	0.054 [0.051–0.056]
Six factors	2981.92	1425	0.831	0.052 [0.049–0.054]
Five factors + acquiescense factor	3098.14	1479	0.824	0.052 [0.049–0.054]
Five factors + acquiescense factor + correlated uniqueness	1984.18	1389	0.935	0.032 [0.029–0.036]

*N = 409. Analyses were run using Mplus 8.7 with orthogonal rotation and the MLR estimator.*

#### Short Versions, Big Five Inventory-2-S, and Big Five Inventory-2-XS

Finally, we examined the psychometric properties of scores from the subsets of items making up the two briefer versions of the BFI-2, that is, the 30-item BFI-2-S and the 15-item BFI-2-XS (Soto and John, 2017b). These two shorter measures were originally designed for use in situations where it is not feasible to administer the full 60-item version. Examples of such situations may be large-scale research projects where personality need to be assessed with fewer questions, and round-robin designs where each participant rates several other participants (Soto and John, 2017b). The 30-item BFI-2-S measures each facet with two items, while the BFI-2-XS measures only the Big Five domains, each assessed with three items (for more information about the development of these brief versions, see Soto and John, 2017b). In the present study, the item scores were obtained by administering the full 60 item BFI-2, thus the results should be considered tentative (Smith et al., 2000).

For both brief versions, an RI-EFA model was specified with five factors, using CF-varimax orthogonal rotation, corresponding to models tested with the brief English-language versions (Soto and John, 2017b). The models were tested in Mplus using the MLR estimator, and good fit was obtained for both models, that is BFI-2-S: χ^2^ (294, *N* = 409) = 602.39, *p* = 0.000, RMSEA = 0.051 [95% C.I. = 0.045–0.056], and CFI = 0.902; and BFI-2-XS: χ^2^ (39, *N* = 409) = 83.06, *p* = 0.000, RMSEA = 0.053 [95% C.I. = 0.037–0.068], and CFI = 0.954. All items had their strongest loading on the intended factor, except for one item in BFI-2-XS (Table 9). The average primary and secondary absolute loadings were 0.72 and 0.13, respectively, for the BFI-2-S; and 0.64 and 0.11, respectively, for the BFI-2-XS. For the BFI-2-S, the reliability of scores was in the range 0.70–0.82 for the domain scales, and 0.27–0.76 for the facet scales (Table 10). Thus, the reliability is very low for some facets, as expected when using only two items per scale. For the BFI-2-XS, the reliability of scores for the domain scales ranged from 0.46 to 0.56. In comparison, Soto and John (2017b) reported corresponding reliabilities of scores in an Internet sample, in the range of 0.74–0.84 for the domain scales and 0.42–0.79 for the facet scales from the BFI-2-S; and 0.49–0.69 for the domain scales from the BFI-2-XS. Thus, in the present sample, the Norwegian BFI-2-S and BFI-2-XS provided scores with comparable structural validity but somewhat lower internal consistency than reported for their English-language counterparts.

**TABLE 9:** Factor loadings from RI-EFA of item scores from the BFI-2-S and BFI-2-XS.

Item	Extraversion	Agreeableness	Conscientiousness	Negative Emotionality	Open-Mindedness
BFI1	**–0.42**/**–0.42**	0.06/–0.03	–0.13/–0.09	0.01/0.03	0.02/0.01
BFI6	**0.66**/**0.62**	0.14/–0.08	–0.04/–0.05	–0.04/–0.03	0.22/0.24
BFI11	**0.60**/**0.57**	–0.21/0.25	–0.07/–0.04	–0.26/–0.28	0.03/0.08
BFI16	**0.56**	–0.27	0.07	–0.07	0.07
BFI21	**–0.54**	–0.09	0.10	0.07	–0.35
BFI26	**–0.27**	0.01	0.24	0.17	–0.02
BFI2	0.11/0.04	**–0.63**/**0.62**	0.07/0.04	0.23/0.18	0.05/–0.01
BFI7	0.21/0.22	**0.51**/**–0.39**	0.28/0.23	0.26/0.25	–0.06/–0.06
BFI12	0.15/0.13	**–0.51**/**0.49**	0.07/0.04	–0.19/–0.23	–0.04/–0.04
BFI17	–0.03	**0.73**	0.10	–0.05	0.01
BFI22	–0.03	–**0.40**	–0.20	–0.06	0.09
BFI27	0.08	**0.50**	0.06	0.33	–0.04
BFI3	–0.01/0.00	0.00/0.01	**0.76**/**0.90**	0.04/0.03	0.12/0.06
BFI8	–0.21/–0.14	0.12/–0.16	**0.41**/**0.36**	0.21/0.23	–0.03/–0.08
BFI13	0.15/0.15	–0.28/**0.29**	**–0.30**/–0.23	–0.08/–0.09	0.08/0.13
BFI18	–0.02	–0.05	**–0.68**	0.06	–0.01
BFI23	0.20	–0.11	**–0.39**	–0.19	0.11
BFI28	0.00	0.11	**0.65**	0.05	–0.08
BFI4	–0.14/–0.13	–0.09/0.11	–0.07/–0.03	**0.73**/**0.69**	0.00/–0.05
BFI9	–0.20/–0.13	0.21/–0.21	0.09/0.09	**0.74**/**0.80**	0.05/–0.02
BFI14	–0.08/–0.07	0.06/–0.14	–0.02/–0.01	**–0.81**/**–0.78**	0.04/0.06
BFI19	0.17	0.10	0.01	**–0.61**	0.07
BFI24	0.36	–0.02	–0.06	**–0.50**	0.11
BFI29	0.34	0.13	0.15	0.56	–0.04
BFI5	–0.04/–0.07	–0.03/0.01	–0.04/0.00	0.06/0.08	**0.58**/**0.49**
BFI10	0.00/0.05	0.01/-0.06	0.02/–0.05	0.10/0.08	**–0.47**/**–0.52**
BFI15	0.31/0.29	0.05/–0.02	0.04/0.10	–0.14/–0.12	**0.65**/**0.68**
BFI20	0.01	0.07	–0.02	–0.10	**–0.56**
BFI25	–0.10	0.02	–0.01	0.25	**0.38**

*Values before the forward slash are for the BFI-2-S, and after the forward slash for the BFI-2-XS. The strongest factor loading for each item is bolded.*

**TABLE 10:** Cronbach’s alpha for scores from BFI-2-S and BFI-2-XS.

Domain and facet scales	BFI-2-S	BFI-2-XS
Extraversion	0.70	0.56
Sociability	0.50	
Assertiveness	0.76	
Energy Level	0.44	
Agreeableness	0.72	0.46
Compassion	0.64	
Respectfulness	0.38	
Trust	0.55	
Conscientiousness	0.72	0.46
Organization	0.70	
Productiveness	0.49	
Responsibility	0.27	
Negative Emotionality	0.82	0.79
Anxiety	0.69	
Depression	0.66	
Emotional Volatility	0.70	
Open-Mindedness	0.73	0.56
Intellectual Curiosity	0.35	
Aesthetic Sensitivity	0.62	
Creative Imagination	0.71	

*N = 409. The coefficients were calculated on subsets of item scores after administering the full 60-item BFI-2.*

## Overall Discussion

The aim of the present research was to assess whether the good psychometric properties of scores from the original, English-language BFI-2 could be replicated with the Norwegian adaptation of BFI-2. The results suggest that the Norwegian adaptation of the BFI-2 provides scores with good psychometric properties, comparable to those reported for the original BFI-2.

Overall, the scores from the Norwegian BFI-2 showed very good structural validity and reliability. In Study 2, the highest factor loading for each item was on the intended factor. The reliability of scores were around 0.80 for the Big Five domains, and 0.60–0.70 for the facets, which is comparable to what has been reported for the original BFI-2 (Soto and John, 2017a). Thus, the Norwegian adaptation of BFI-2 seems to be well suited for use in research. One should be careful, however, in using the BFI-2 to assess personality traits on the individual level, particularly when measuring facets. Due to low reliability of facet scores, some scores will likely have a high standard error of measurement, leading to imprecise scores. Nunnally (1978) recommended that scales providing scores with reliability of 0.70 might be useful in early stages of developing a questionnaire, while 0.80 might be adequate for many purposes in basic research (for a discussion, see Gugiu and Gugiu, 2018). Thus, the BFI-2 may be a useful tool for researchers who need a brief measure of the Big Five personality domains along with narrow traits within the Big Five.

The present research has several strengths. A thorough translation process was conducted, and the final adaptation of BFI-2 was refined across two samples. Moreover, multilevel modeling was used to assess convergent and divergent validity with others’ ratings of Big Five. The present research, however, also has some limitations that may be addressed in future studies.

One limitation is that only the BFI (and the brief version BFI-20) were used to provide evidence for convergent and divergent validity with alternative measures of the Big Five. This is not optimal, as the BFI is a precursor for the BFI-2, and about one-third of the BFI-2 items are identical to items in the BFI. Despite this limitation, however, the overall evidence provides support for the validity of scores from the Norwegian adaptation of the BFI-2. First, the psychometric analyses of the scores in the present study (i.e., PCA, CFA, reliability analysis, and congruence analysis) clearly demonstrate that the Norwegian adaptation of BFI-2 provide scores that are highly similar to the scores provided by the original BFI-2. Second, the present study shows that the scores correlate in expected ways with both self- and other ratings on the BFI/BFI-20, and in expected ways with empathic concern and perspective taking, supporting the construct validity of scores. Nevertheless, future studies may further assess the convergent and divergent validity of scores by relating them to scores from other established personality inventories in Norwegian, preferably inventories that measure personality facets. Moreover, future studies should also try to replicate these findings in larger and more diverse samples and assess predictive validity of facet scores in relation to other important outcomes. Finally, some items and scales in the BFI-2 seem to provide high scores, and future studies might look closer at whether this is due to social desirability or other issues.

## Conclusion

The present research supports the validity and reliability of scores from the Norwegian adaptation of the BFI-2. The psychometric properties are good, and comparable to those reported for scores from the original, English-language version. The Norwegian adaptation of the BFI-2 may be useful for personality research in both Norwegian-language and cross-cultural contexts, when one needs to measure the Big Five or more narrow personality traits.

## Data Availability Statement

The raw data supporting the conclusions of this article will be made available by the authors, without undue reservation.

## Ethics Statement

Ethical review and approval was not required for the study on human participants in accordance with the local legislation and institutional requirements. The patients/participants provided written or active informed consent to participate in this study.

## Author Contributions

HF collected data, organized the database, performed the statistical analyses, and wrote the first draft of the manuscript. CS provided syntax for SPSS analyses. Both authors contributed to the development and refinement of the Norwegian adaptation of the BFI-2, interpretation of the results, manuscript revision, read, and approved the submitted version.

## Conflict of Interest

The authors declare that the research was conducted in the absence of any commercial or financial relationships that could be construed as a potential conflict of interest.

## Publisher’s Note

All claims expressed in this article are solely those of the authors and do not necessarily represent those of their affiliated organizations, or those of the publisher, the editors and the reviewers. Any product that may be evaluated in this article, or claim that may be made by its manufacturer, is not guaranteed or endorsed by the publisher.
