# Supplementary material for: The Norwegian Adaptation of the Big Five Inventory-2
Source: Front Psychol. 2022 May 18;13:858920. doi: 10.3389/fpsyg.2022.858920 (PMC9158541; doi:10.3389/fpsyg.2022.858920)
Supplement: Supplementary file 1 [file Table_1.pdf]

Supplementary Table 1

Factor loadings from RI-EFA of item scores from the BFI-2

Item	E	A	C	N	O	ARS
BFI1	**.60**	-.24	.07	-.01	.01	.11
BFI6	**.59**	.23	-.05	-.29	.11	.10
BFI11	**-.48**	.35	.05	-.09	-.06	.10
BFI16	**-.50**	.03	-.11	-.03	.09	.11
BFI21	**.66**	.21	-.03	.01	.10	.10
BFI26	**-.23**	-.04	**.23**	.16	-.01	.10
BFI31	**-.62**	.05	.05	.10	-.02	.08
BFI36	**-.41**	-.05	-.02	.11	-.12	.11
BFI41	**.59**	-.15	-.08	-.19	-.02	.11
BFI46	**.65**	-.20	.02	.14	.00	.10
BFI51	**-.49**	-.16	.09	.03	-.25	.10
BFI56	**.56**	-.25	.01	.06	.11	.11
BFI2	.10	**-.68**	.09	.23	.04	.12
BFI7	-.07	**-.39**	-.19	-.06	.08	.20
BFI12	.11	**.45**	.03	.33	-.08	.10
BFI17	-.08	**.60**	-.07	-.06	-.06	.10
BFI22	.34	**.36**	.16	.16	.06	.10
BFI27	.08	**-.41**	.06	-.20	.09	.10
BFI32	.10	**-.47**	-.10	-.01	.13	.14
BFI37	.23	**.46**	.27	.25	-.05	.09
BFI42	-.08	**.39**	.02	.34	.11	.10
BFI47	-.04	**.70**	.08	-.06	.02	.09
BFI52	-.06	**-.46**	-.09	-.08	.02	.19
BFI57	.16	**-.52**	.07	-.19	-.04	.10
BFI3	.01	-.10	**.75**	-.01	.09	.09
BFI8	-.14	.05	**.54**	.13	.01	.09
BFI13	.00	-.18	**-.46**	-.01	-.05	.16
BFI18	-.03	.08	**-.73**	.21	-.01	.10
BFI23	-.20	.02	**.45**	.15	.01	.10

BFI28	.04	.01	**.64**	.02	-.09	.10
BFI33	.00	.03	**-.73**	.12	.02	.10
BFI38	.37	.03	**-.46**	-.07	.01	.12
BFI43	.11	-.23	**-.34**	-.07	.07	.17
BFI48	.03	.06	**.56**	-.06	-.01	.10
BFI53	.16	-.02	**-.43**	-.16	.07	.12
BFI58	.11	.14	**.61**	.11	.01	.09
BFI4	.08	.15	.05	**-.60**	.05	.09
BFI9	.16	-.11	-.04	**-.49**	.10	.10
BFI14	.15	.09	.08	**.66**	-.07	.08
BFI19	-.07	.00	-.07	**.63**	-.03	.10
BFI24	.33	.06	-.05	**-.50**	.09	.10
BFI29	-.17	.08	.04	**-.84**	.03	.08
BFI34	-.07	-.10	-.10	**.74**	.02	.08
BFI39	-.10	.09	.02	**.81**	.13	.09
BFI44	-.09	.08	-.07	**-.67**	.05	.10
BFI49	.13	.16	.08	**-.66**	.02	.08
BFI54	-.14	.17	.04	**.77**	.09	.08
BFI59	.37	.13	.11	**.56**	-.04	.08
BFI5	.08	.08	-.04	-.08	**-.56**	.08
BFI10	.19	-.15	.03	-.03	**.46**	.12
BFI15	.16	.15	-.15	-.15	**.54**	.10
BFI20	-.13	-.02	-.01	.05	**.69**	.08
BFI25	.01	-.12	-.02	.11	**-.38**	.09
BFI30	-.09	.03	-.06	.04	**-.62**	.09
BFI35	-.04	-.06	.01	.11	**.68**	.09
BFI40	-.13	.02	.01	.23	**.44**	.10
BFI45	-.04	.12	.00	.01	**-.36**	.12
BFI50	.03	.16	.01	-.12	**-.57**	.07
BFI55	.05	.00	-.02	.12	**-.50**	.10
BFI60	.26	.09	.05	-.12	**.59**	.10

Note. E = Extraversion, A = Agreeableness, C = Conscientiousness, N = Negative Emotionality, O = Open-Mindedness, and ARS = Acquiescent Response Style. The strongest factor loading for each item is bolded.
